# The Effects of Cardiometabolic Factors on the Association Between Serum Uric Acid and Chronic Kidney Disease in Chinese Middle-Aged and Older Population: A Mediation Analysis

**DOI:** 10.3389/fendo.2021.702138

**Published:** 2021-06-18

**Authors:** Lu Xu, Hang Sun, Lili Liu, Siyan Zhan, Shengfeng Wang, Xiaozhen Lv, Yongfeng Song

**Affiliations:** ^1^ Department of Epidemiology and Biostatistics, School of Public Health, Peking University, Beijing, China; ^2^ Department of Endocrinology, Shandong Provincial Hospital, Cheeloo College of Medicine, Shandong University, Jinan, China; ^3^ Shandong Clinical Medical Center of Endocrinology and Metabolism, Jinan, China; ^4^ Institute of Endocrinology and Metabolism, Shandong Academy of Clinical Medicine, Jinan, China; ^5^ Research Center of Clinical Epidemiology, Peking University Third Hospital, Beijing, China; ^6^ Center for Intelligent Public Health, Institute for Artificial Intelligence, Peking University, Beijing, China; ^7^ Beijing Dementia Key Lab, National Clinical Research Center for Mental Disorders, Peking University Sixth Hospital (Institute of Mental Health), Beijing, China; ^8^ Shandong Institute of Endocrine & Metabolic Diseases, Jinan, China; ^9^ Department of Endocrinology, Shandong Provincial Hospital Affiliated to Shandong First Medical University, Jinan, China

**Keywords:** mediation analysis, serum uric acid, chronic kidney disease, cardiometabolic factors, dyslipidemia, hypertension, hyperglycemia

## Abstract

**Introduction:**

To explore whether dyslipidemia, hyperglycemia or hypertension has mediating effect on the association between serum uric acid (SUA) and the development of chronic kidney disease (CKD).

**Methods:**

We conducted a mediation analysis to explore the potential mediating effects of systolic blood pressure (SBP), diastolic blood pressure (DBP), blood glucose, triglyceride (TG), total cholesterol (TC), high-density lipoprotein cholesterol (HDL-C) and low-density lipoprotein cholesterol (LDL-C) on the association between SUA and estimated glomerular filtration rate (eGFR). The data were obtained from China Health and Retirement Longitudinal Study (CHARLS), covering 5,762 individuals.

**Results:**

SUA had a negative dose-response total effect on eGFR (*β* -3.11, 95% CI -3.40 to -2.82, *P*-value<0.001). The linear regression between SUA and seven potential mediators indicated that blood glucose (*β* 0.80, 95% CI 0.18 to 1.42, *P*-value=0.012), TG (*β* 10.01, 95% CI 8.22 to 11.79, *P-*value<0.001), TC (*β* 2.64, 95% CI 1.83 to 3.45, *P*-value<0.001), HDL-C (*β* -0.27, 95% CI -0.52 to -0.02, *P-*value=0.034) and LDL-C (*β* 1.15, 95% CI 0.49 to 1.80, *P*-value=0.001) all had significant dose-response association with SUA, but SBP and DBP showed no significant association with SUA. In terms of the association between potential mediators and eGFR, only TG (*β* 0.003, 95% CI -0.001 to 0.01, *P*-value=0.117) and HDL-C (*β* 0.01, 95% CI -0.02 to 0.04, *P*-value=0.444) did not have significant linear association with eGFR. The linear regression showed that SUA was directly associated with eGFR (*P-*value<0.001).

**Conclusions:**

This study supported that the association between SUA and the risk of CKD was not mediated by hypertension, hyperglycemia or dyslipidemia.

## Introduction

Chronic kidney disease (CKD), characterized by ongoing and irreversible damage of the renal parenchyma which leads to chronic deterioration of renal function (1), is mainly reflected by decline of estimated glomerular filtration rate (eGFR) (2). CKD has been recognized as a rapidly growing worldwide public health problem (3), especially in developing countries (3, 4).

In recent years, serum uric acid (SUA), the end product of purine metabolism in humans (5), has gradually been considered as a risk factor of CKD (6–11). There are many potential mechanisms behind this, such as the activation of the renin-angiotensin system (RAS) (12, 13), the proliferation of the vascular smooth muscle cells (VSMC) through Cyclooxygenase-2 (COX-2) dependent pathway (13), and direct fibrogenic effect on renal cells (13).

Existing evidence has suggested that elevated SUA concentration may play a role in the development of CKD (7, 8), which may be mediated by cardiometabolic factors. SUA has been reported to be associated with the pathogenesis of dyslipidemia (7), diabetes (14) and hypertension (15) which are also the risk factors of CKD (16), and these risk factors usually coexist and could influence each other (17). However, limited studies have examined the mediating effect of such cardiometabolic factors on the association between SUA and CKD; therefore, whether dyslipidemia, hyperglycemia or hypertension has mediating effect on the association between SUA and the development of CKD remains unclear.

This population-based study used nationally representative survey data to explore whether hyperglycemia, hypertension or dyslipidemia has mediating effect on the association between SUA and CKD in Chinese middle-aged and older population.

## Methods

### Database and Study Population

The China Health and Retirement Longitudinal Study (CHARLS) was a nationally representative longitudinal survey among the population aged 45 years and older in China. This survey was carried out every two or three years. To date, there have been four surveys conducted in 2011 (visit 1), 2013 (visit 2), 2015 (visit 3) and 2018 (visit 4), respectively. Blood sample data were collected at visit 1 and visit 3. Detailed information about this survey is available elsewhere (18). This study was approved by the Ethical Review Committee of Peking University (IRB00001052-11015), and written informed consent was obtained from each participant.

Participants with available blood sample data at visit 1 were included in this study. We excluded the participants younger than 45 years old at baseline, those were not followed at visit 3, those lacked SUA data at visit 1 or creatine data at visit 3, and those did not have a blood test in fasting state at visit 1 or visit 3. Participants with hypouricemia [i.e., SUA < 2 mg/dL for both sexes (19)] at baseline were excluded as well.

### Exposure and Outcome Assessment

The exposure variable was baseline SUA. In this study, the outcome considered in this study was eGFR (mL/min per 1.73 m^2^) which was estimated using the CKD-EPI creatinine equation (2009): 141 × min(Scr/κ, 1)α×max(Scr/κ, 1)-1.209×0.993Age[×1.018 if female][×1.159 if black], where Scr is serum creatinine, κ is 0.7 for females and 0.9 for males, α is -0.329 for females and -0.411 for males, min is the minimum of Scr/κ or 1, and max is the maximum of Scr/κ or 1 (20).

### Covariate Assessment

The covariates considered in this study were as follows: age (years), sex (male, female), smoking (never, current/former), drinking (never, current/former), body mass index (BMI, kg/m^2^) (21, 22), and medication use (i.e., medication treatment for hypertension, hyperglycemia and dyslipidemia). BMI was calculated by dividing weight (kg) by the square of height (m) and BMI was categorized into four levels (underweight: <18.5 kg/m^2^, normal: ≥18.5–24 kg/m^2^, overweight: ≥24–28 kg/m^2^, obesity: ≥28 kg/m^2^ (23).

### Potential Mediators

The selected potential mediators were systolic blood pressure (SBP), diastolic blood pressure (DBP),blood glucose, triglyceride (TG), total cholesterol (TC), high-density lipoprotein cholesterol (HDL-C) and low-density lipoprotein cholesterol (LDL-C), since previous studies reported the association between SUA with hypertension, hyperglycemia or dyslipidemia (7, 14, 15) and the association between hypertension, hyperglycemia or dyslipidemia with CKD (16). All potential mediators was measured at visit 3.

### Statistical Analysis

Comparisons of baseline demographic characteristics or clinical features between sexes were performed by Student t test for continuous variables, and Pearson chi-squared test for categorical variables.

Before examination of the possible mediating effects of the seven potential mediators on the association between SUA and eGFR, we evaluated the total effect of SUA on eGFR over five-year period. A multivariable linear regression model adjusted for age, sex, smoking, drinking and BMI level (underweight: <18.5 kg/m^2^, normal: ≥18.5–24 kg/m^2^, overweight: ≥24–28 kg/m^2^, obesity: ≥28 kg/m^2^) was used, with SUA analyzed as a continuous variable.

We followed standard procedures for mediation analysis, using three main steps to do a series of linear regressions adjusted for age, sex, smoking, drinking, BMI level and medication treatment for hypertension, hyperglycemia or dyslipidemia (24). In the first step, the association between SUA and a range of potential mediators was examined. In the second step, the effect of each potential mediator on eGFR was evaluated. In the third step, the potential mediators and baseline SUA were all included in linear regression to examine whether SUA has a direct or indirect effect on eGFR. The indirect effect between SUA and eGFR caused by the potential mediators was evaluated using khb program in Stata version 15.0 (25). Subgroup analyses by sex (male, female) were performed.

The inverse probability weighting method was adopted to take non-response rate into consideration. The individuals with missing data in some variables were not considered in the analyses including the corresponding variables. All statistical analyses were performed by Stata version 15.0 (StataCorp, College Station, TX, USA). Two-sided *P-*value less than 0.05 was set as the statistically significant level.

## Results

### Baseline Characteristics

This study included 5,762 participants, with 3,132 females and 2,630 males. Males were more likely to be older, smokers, drinkers, lower in BMI, TG, TC, LDL-C and eGFR, and, higher in SUA (**Table 1**). There were no significant differences in SBP, DBP, blood glucose, and HDL-C between two sexes.

**Table 1:** Characteristics of participants at baseline.

Characteristics	Overall	Female	Male	*P*-value
**Participants, n (%)**	5,762	3,132	2,630	
**Age (years, SD)**	58.84 (9.13)	58.43 (9.23)	59.31 (8.98)	<0.001
**Smoking, n (%)**				<0.001
Current/former	2,209 (37.84)	237 (7.16)	1,972 (73.12)	
**Drinking, n (%)**				<0.001
Current/former	2,201 (38.99)	455 (13.52)	1,746 (68.29)	
**BMI (kg/m^2^), n (%)**				<0.001
Underweight (<18.5 kg/m^2^)	326 (5.21)	169 (4.70)	157 (5.79)	
Normal (≥18.5–24 kg/m^2^)	2,905 (48.69)	1,404 (42.65)	1,501 (55.61)	
Overweight (≥24–28 kg/m^2^)	1,754 (32.79)	1,044 (36.67)	710 (28.34)	
Obesity (≥28 kg/m^2^)	753 (13.31)	499 (15.98)	254 (10.26)	
**SUA (mg/dL, SD)**	4.53 (1.31)	4.11 (1.06)	5.02 (1.39)	<0.001
**SBP (mmHg)**	130.65 (20.92)	131.07 (22.07)	130.15 (19.51)	0.118
**DBP (mmHg)**	76.06 (11.92)	75.91 (11.86)	76.22 (11.99)	0.359
**Blood glucose (mg/dL, SD)**	108.86 (33.41)	108.51 (31.55)	109.26 (35.42)	0.396
**TG (mg/dL, SD)**	131.03 (87.96)	137.37 (87.72)	123.75 (87.70)	<0.001
**TC (mg/dL, SD)**	192.15 (38.46)	196.76 (38.41)	186.84 (37.83)	<0.001
**HDL-C (mg/dL, SD)**	49.57 (15.17)	49.90 (14.55)	49.19 (15.85)	0.073
**LDL-C (mg/dL, SD)**	116.28 (34.68)	119.30 (34.77)	112.80 (34.25)	<0.001
**eGFR (mL/min per 1.73 m^2^, SD)**	92.34 (14.55)	92.69 (14.47)	91.93 (14.62)	<0.050

SD, standard deviation; BMI, body mass index; SUA, serum uric acid; SBP, systolic blood pressure; DBP, diastolic blood pressure; TG, triglyceride; TC, total cholesterol; HDL-C, high-density lipoprotein cholesterol; LDL-C, low-density lipoprotein cholesterol; eGFR, estimated glomerular filtration rate.

There were 20, 21, 24, 724, 724, 10, 29, 5, 13 and 11 individuals with missing information in smoking, drinking, BMI, SBP, DBP, blood glucose, TG, TC, LDL-C and eGFR, respectively.

### Total Effect of SUA on eGFR

After adjustment for age, sex, smoking, drinking and BMI level, there was a negative dose-response relationship of SUA and eGFR (*β* -3.11, 95% CI -3.40 to -2.82, **Table 2**). Repeating the linear regressions in different sexes, such a dose-response relationship was still significant in males (*β* -2.71, 95% CI -3.09 to -2.34, **Table 3**) and females (*β* -3.67, 95% CI -4.14 to -3.21, **Table 4**).

**Table 2:** The mediating effects of cardiometabolic factors on the association between SUA and eGFR.

Step	Effect	*β* (95% CI)	*P*-value
Total effect: association between SUA and eGFR			
	SUA	**-3.11 (-3.40 to -2.82)**	**<0.001**
Mediation analysis			
Step 1: association between SUA and potential mediators			
	SBP	-0.25 (-0.64 to 0.14)	0.214
	DBP	0.02 (-0.20 to 0.25)	0.841
	Blood glucose	**0.80 (0.18 to 1.42)**	**0.012**
	TG	**10.01 (8.22 to 11.79)**	**<0.001**
	TC	**2.64 (1.83 to 3.45)**	**<0.001**
	HDL-C	**-0.27 (-0.52 to -0.02)**	**0.034**
	LDL-C	**1.15 (0.49 to 1.80)**	**0.001**
Step 2: association between potential mediators and eGFR			
	SBP	**0.05 (0.03 to 0.07)**	**<0.001**
	DBP	**0.06 (0.03 to 0.10)**	**<0.001**
	Blood glucose	**0.03 (0.01 to 0.04)**	**<0.001**
	TG	0.003 (-0.001 to 0.01)	0.117
	TC	**-0.01 (-0.02 to -0.0001)**	**0.047**
	HDL-C	0.01 (-0.02 to 0.04)	0.444
	LDL-C	**-0.02 (-0.03 to -0.01)**	**<0.001**
Step 3 (direct effect): association between SUA and eGFR that excluded the effects of potential mediators			
	SBP	**-3.05 (-3.35 to -2.76)**	**<0.001**
	DBP	**-3.07 (-3.36 to -2.77)**	**<0.001**
	Blood glucose	**-3.14 (-3.43 to -2.85)**	**<0.001**
	TG	**-3.20 (-3.49 to -2.90)**	**<0.001**
	TC	**-3.09 (-3.39 to -2.80)**	**<0.001**
	HDL-C	**-3.10 (-3.39 to -2.80)**	**<0.001**
	LDL-C	**-3.08 (-3.37 to -2.78)**	**<0.001**
Indirect effect (caused by each of the mediators)			
	SBP	-0.01 (-0.05 to 0.02)	0.509
	DBP	0.002 (-0.03 to 0.03)	0.918
	Blood glucose	0.02 (-0.01 to 0.06)	0.109
	TG	**0.10 (0.03 to 0.17)**	**0.003**
	TC	-0.003 (-0.05 to 0.04)	0.856
	HDL-C	-0.001 (-0.01 to 0.01)	0.878
	LDL-C	-0.02 (-0.05 to 0.01)	0.158

SUA, serum uric acid; eGFR, estimated glomerular filtration rate; SBP, systolic blood pressure; DBP, diastolic blood pressure; TG, triglyceride; TC, total cholesterol; HDL-C, high-density lipoprotein cholesterol; LDL-C, low-density lipoprotein cholesterol.

All logistic regressions were adjusted for age, sex, smoking, drinking, medication treatment for hypertension, diabetes or dyslipidemia, and body mass index level. The bold values represent statistically significant effects.

**Table 3:** The mediating effects of cardiometabolic factors on the association between SUA and eGFR in males.

Step	Effect	*β* (95% CI)	*P*-value
Total effect: association between SUA and eGFR			
	SUA	**-2.71 (-3.09 to -2.34)**	**<0.001**
Mediation analysis			
Step 1: association between SUA and potential mediators			
	SBP	-0.03 (-0.54 to 0.48)	0.898
	DBP	0.20 (-0.10 to 0.51)	0.184
	Blood glucose	**0.90 (0.10 to 1.70)**	**0.028**
	TG	**6.41 (4.21 to 8.61)**	**<0.001**
	TC	**2.50 (1.50 to 3.51)**	**<0.001**
	HDL-C	0.07 (-0.27 to 0.42)	0.675
	LDL-C	**1.40 (0.56 to 2.24)**	**0.001**
Step 2: association between potential mediators and eGFR			
	SBP	0.02 (-0.01 to 0.05)	0.107
	DBP	0.03 (-0.02 to 0.08)	0.179
	Blood glucose	**0.02 (0.003 to 0.04)**	**0.023**
	TG	0.01 (-0.0002 to 0.01)	0.059
	TC	**-0.02 (-0.03 to -0.003)**	**0.021**
	HDL-C	0.004 (-0.04 to 0.05)	0.844
	LDL-C	**-0.04 (-0.05 to -0.02)**	**<0.001**
Step 3 (direct effect): association between SUA and eGFR that excluded the effects of potential mediators			
	SBP	**-2.69 (-3.07 to -2.32)**	**<0.001**
	DBP	**-2.70 (-3.08 to -2.33)**	**<0.001**
	Blood glucose	**-2.76 (-3.13 to -2.38)**	**<0.001**
	TG	**-2.78 (-3.15 to -2.40)**	**<0.001**
	TC	**-2.68 (-3.06 to -2.30)**	**<0.001**
	HDL-C	**-2.70 (-3.08 to -2.32)**	**<0.001**
	LDL-C	**-2.66 (-3.03 to -2.28)**	**<0.001**
Indirect effect (caused by each of the mediators)			
	SBP	-0.001 (-0.02 to 0.02)	0.941
	DBP	0.01 (-0.02 to 0.04)	0.533
	Blood glucose	0.02 (-0.02 to 0.06)	0.229
	TG	**0.08 (0.01 to 0.15)**	**0.035**
	TC	-0.02 (-0.07 to 0.04)	0.490
	HDL-C	0.001 (-0.004 to 0.01)	0.841
	LDL-C	-0.04 (-0.09 to 0.01)	0.112

SUA, serum uric acid; eGFR, estimated glomerular filtration rate; SBP, systolic blood pressure; DBP, diastolic blood pressure; TG, triglyceride; TC, total cholesterol; HDL-C, high-density lipoprotein cholesterol; LDL-C, low-density lipoprotein cholesterol.

All logistic regressions were adjusted for age, sex, smoking, drinking, medication treatment for hypertension, diabetes or dyslipidemia, and body mass index level. The bold values represent statistically significant effects.

**Table 4:** The mediating effects of cardiometabolic factors on the association between SUA and eGFR in females.

Step	Effect	*β* (95% CI)	*P*-value
Total effect: association between SUA and eGFR			
	SUA	**-3.67 (-4.14 to -3.21)**	**<0.001**
Mediation analysis			
Step 1: association between SUA and potential mediators			
	SBP	**-0.63 (-1.24 to -0.01)**	**0.046**
	DBP	-0.24 (-0.59 to 0.11)	0.181
	Blood glucose	0.51 (-0.47 to 1.49)	0.306
	TG	**14.72 (11.8 to 17.63)**	**<0.001**
	TC	**2.85 (1.53 to 4.16)**	**<0.001**
	HDL-C	**-0.68 (-1.05 to -0.31)**	**<0.001**
	LDL-C	0.79 (-0.26 to 1.83)	0.142
Step 2: association between potential mediators and eGFR			
	SBP	**0.08 (0.05 to 0.10)**	**<0.001**
	DBP	**0.09 (0.04 to 0.14)**	**<0.001**
	Blood glucose	**0.03 (0.01 to 0.05)**	**<0.001**
	TG	0.002 (0.004 to 0.01)	0.537
	TC	-0.005 (-0.02 to 0.01)	0.471
	HDL-C	0.02 (-0.03 to 0.06)	0.475
	LDL-C	-0.01 (-0.03 to 0.01)	0.208
Step 3 (direct effect): association between SUA and eGFR that excluded the effects of potential mediators			
	SBP	**-3.58 (-4.05 to -3.11)**	**<0.001**
	DBP	**-3.60 (-4.07 to -3.12)**	**<0.001**
	Blood glucose	**-3.69 (-4.16 to -3.23)**	**<0.001**
	TG	**-3.82 (-4.29 to -3.35)**	**<0.001**
	TC	**-3.68 (-4.15 to -3.21)**	**<0.001**
	HDL-C	**-3.68 (-4.15 to -3.21)**	**<0.001**
	LDL-C	**-3.67 (-4.14 to -3.20)**	**<0.001**
Indirect effect (caused by each of the mediators)			
	SBP	-0.04 (-0.14 to 0.05)	0.372
	DBP	-0.02 (-0.07 to 0.04)	0.494
	Blood glucose	0.02 (-0.03 to 0.06)	0.448
	TG	**0.14 (0.03 to 0.26)**	**0.016**
	TC	0.01 (-0.05 to 0.07)	0.809
	HDL-C	0.004 (-0.04 to 0.05)	0.863
	LDL-C	-0.01 (-0.03 to 0.02)	0.622

SUA, serum uric acid; eGFR, estimated glomerular filtration rate; SBP, systolic blood pressure; DBP, diastolic blood pressure; TG, triglyceride; TC, total cholesterol; HDL-C, high-density lipoprotein cholesterol; LDL-C, low-density lipoprotein cholesterol.

All logistic regressions were adjusted for age, sex, smoking, drinking, medication treatment for hypertension, diabetes or dyslipidemia, and body mass index level. The bold values represent statistically significant effects.

### Test of Mediation

**Figure 1:**
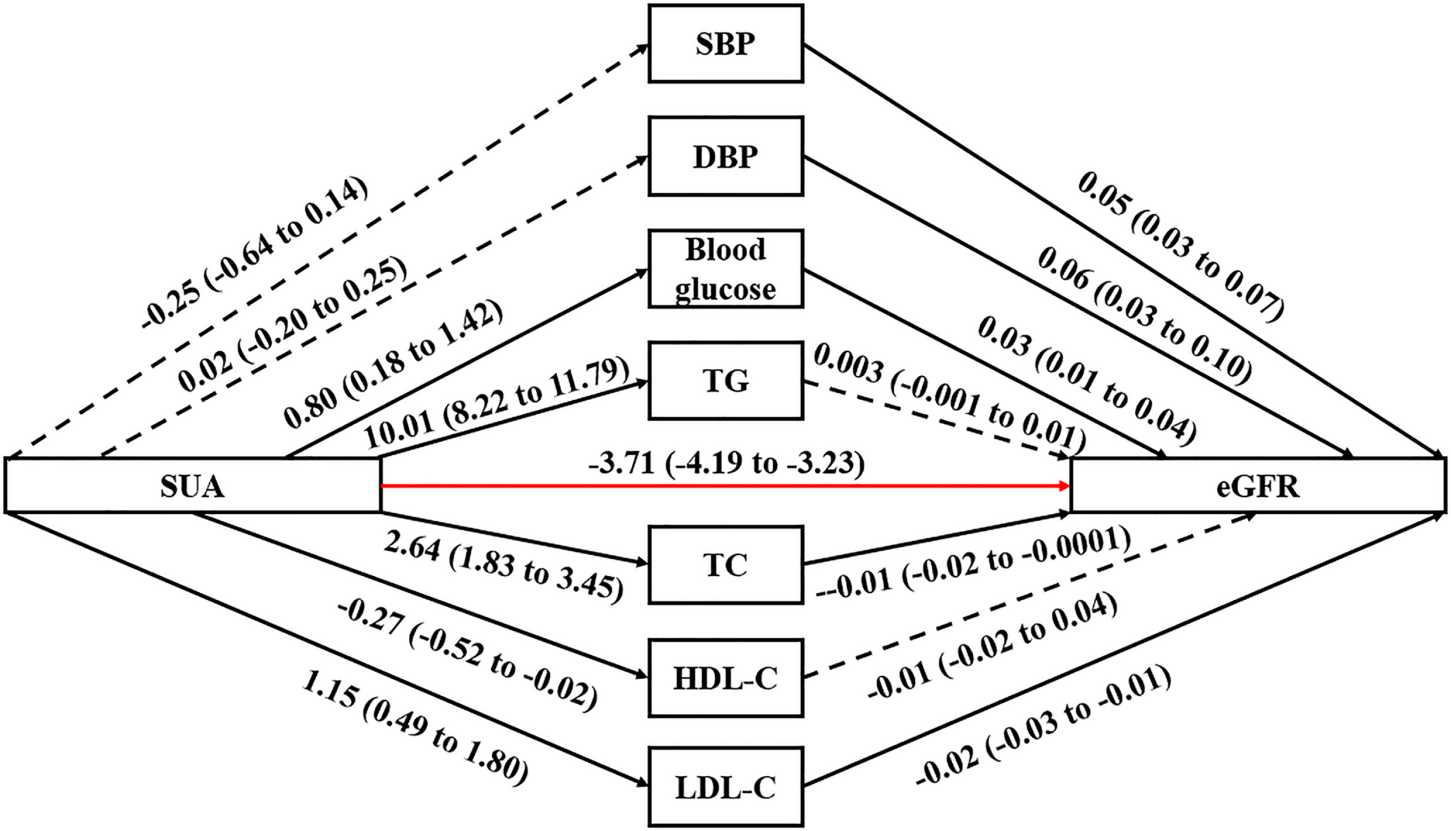
Path diagram for mediational model. SUA, serum uric acid; eGFR, estimated glomerular filtration rate; SBP, systolic blood pressure; DBP, diastolic blood pressure; TG, triglyceride; TC, total cholesterol; HDL-C, high-density lipoprotein cholesterol; LDL-C, low-density lipoprotein cholesterol. The solid arrows represent a significant effect, and the dashed arrow represents a nonsignificant effect. The red arrow represents the association between SUA and eGFR that excluded the effects of the seven potential mediators. The regression linear coefficient *β* and 95% CIs are positioned beside each arrow.

Evaluating the association between SUA and seven potential mediators, blood glucose (*β* 0.80, 95% CI 0.18 to 1.42), TG (*β* 10.01, 95% CI 8.22 to 11.79), TC (*β* 2.64, 95% CI 1.83 to 3.45) and LDL-C (*β* 1.15, 95% CI 0.49 to 1.80) all had significant positive dose-response relationship with SUA, while HDL-C had negative dose-response relationship with SUA (*β* -0.27, 95% CI -0.52 to -0.02, **Table 2** and **Figure 1**).

In terms of the association between potential mediators and eGFR, only TG (*β* 0.003, 95% CI -0.001 to 0.01) and HDL-C (*β* 0.01, 95% CI -0.02 to 0.04) did not have significant linear association with eGFR. There were significant positive dose-response relationships between SBP (*β* 0.05, 95% CI 0.03 to 0.07), DBP (*β* 0.06, 95% CI 0.03 to 0.10), blood glucose (*β* 0.03, 95% CI 0.01 to 0.04) and eGFR. Also, there were significant negative dose-response relationships between TC (*β* -0.01, 95% CI -0.02 to -0.0001) or LDL-C (*β* -0.02, 95% CI -0.03 to -0.01) and eGFR.

The linear regression including both the potential mediators and SUA showed that SUA was directly associated with eGFR (*P-*value<0.001). However, except for TG, the indirect effects of other potential mediators were all non-significant. Although the indirect effect of TG was significant, it was opposite to the total effect; therefore, TG was not a mediator of the association between SUA and eGFR. Stratified by different sexes, similar results were observed (**Tables 3** and **4**).

## Discussion

This national population-based study supported a direct association between SUA and the development of CKD, with no mediating effect of dyslipidemia, hypertension or hyperglycemia. The potential mechanisms for the direct effect of SUA are as follows. First, RAS would be activated by high-level SUA (26), thereby increasing the glomerular pressure and generating direct fibrogenic effect on renal cells which could lead to kidney disease (27). Second, an animal study indicated SUA could stimulate the proliferation of VSMC by uric acid-mediated COX-2 dependent pathway, thereby inducing preglomerular vasculopathy, vascular injury and renal dysfunction (28). Third, SUA probably had a direct effect on renal tubular cells through the induction of phenotypic transition of cultured renal tubular cells (i.e., epithelial-to-mesenchymal transition, EMT) (29), and EMT is an important contributor to the pathogenesis of renal fibrosis (30). Fourh, SUA may also induce CKD *via* the decrease of NO production and induction of oxidative stress (28).

As hypertension, hyperglycemia and dyslipidemia are risk factors of CKD (16) and also related to high-level SUA (7), it is possible that the association between SUA and CKD could be explained by the mediating effect of these cardiometabolic risk factors. However, the result of the mediation analysis indicated that there was no mediating effect of hypertension, hyperglycemia or dyslipidemia on the association between SUA and CKD. The possible explanations are as follows. First, although high-level SUA could increase the risk of hypertension, hyperglycemia and dyslipidemia, the strong direct damage effect of SUA on CKD may be more potent than the effect of hypertension, hyperglycemia or dyslipidemia on renal function in the initial stage of CKD. Also, the sample size in our study can ensure us to exclude the contribution of each mediator to the indirect effect, which only made up less than 5% of the total effect. Second, the marker of early renal damage from hyperglycemia and hypertension is microalbuminuria (31, 32), and only with disease progressing, high-level blood pressure and high-level blood glucose could cause obvious damage to eGFR. The study period in our study was only five years; therefore, the effect of hypertension or hyperglycemia on the decline of eGFR may be weak in the initial stage of CKD.

The significant relationship between SUA and the development of hyperglycemia observed in this study was consistent with previous studies (14, 33, 34). The positive association between SUA and hyperglycemia can be explained by nitric oxide reduction induced by hyperuricemia (35). The decrease of nitric oxide lowers insulin-stimulated glucose intake in skeletal muscle and prompts insulin resistance (36), thereby leading to hyperglycemia. The association between SUA and the development of dyslipidemia found in this study was also consistent with some previous studies (7, 34). However, other studies indicated that there was no relationship between SUA and the development of dyslipidemia (37, 38). Therefore, the role of SUA in the pathogenesis of dyslipidemia is still controversial and future work in this regard is warranted.

We also observed the positive relationships between SBP, DBP or blood glucose and eGFR. This phenomenon could be explained by glomerular hyperfiltration in initial stage of hypertension and hyperglycemia (13, 39, 40), since the glomerular hyperfiltration in those with hyperglycemia and hypertension may be caused by improper vasodilation of afferent arteriole (39) and increased glomerular hydraulic pressure, respectively (41). It was noticeable that there was no significant dose-response relationship between HDL-C and eGFR. One study suggested that lower HDL-C was related to higher eGFR in individuals without kidney disease (42). One explanation is that individuals with high-level HDL-C may also have high-level TC and high-level LDL-C which are also negatively associated with eGFR as observed in our study and other previous studies (43, 44). Therefore, HDL-C may not have a protective effect on kidney function. However, another study reported that HDL-C was critical for the protection against renal dysfunction (45). Also, it was found that high-level HDL-C was not related to reduced mortality risk in individuals with kidney dysfunction (46). These conflicting results probably indicated that the effect of HDL-C could be heterogeneous; therefore, the mechanisms of how HDL-C influence the development of CKD remains unclear.

This longitudinal study utilized the nationally representative data to explore whether SUA has a direct effect on the development of CKD among Chinese middle-aged and older population. But this study still has limitations. First, no data on albuminuria were included, which is an important factor for the definition of CKD. However, the definition of CKD using eGFR < 60 mL/min per 1.73 m^2^ is well-accepted and acknowledged in population-based studies (47, 48). Second, in CHARLS, the identification of hyperglycemia and hypertension depended on not only the data from blood test and physical examination, but also self-reported physician diagnosis. But according to previous validation studies, the self-reports of common chronic diseases were accurate and well-accepted (49, 50). In addition, many published high-quality studies based on CHARLS also used such self-reported physician diagnosis, which confirmed the reliability and accuracy of the data.

## Conclusions

This study supported that the association between SUA and the risk of CKD was not mediated by hypertension, hyperglycemia or dyslipidemia. These findings highlight the important role of SUA as a risk factor for CKD. Therefore, it is necessary to regularly measure SUA in order to circumvent the manifestation of CKD and its progression into end-stage renal disease.

## Data Availability Statement

The raw data supporting the conclusions of this article will be made available by the authors, without undue reservation.

## Ethics Statement

This study was approved by the Ethical Review Committee of Peking University (IRB00001052-11015). The patients/participants provided their written informed consent to participate in this study.

## Author Contributions

SW, XL, and YS conceived and designed the study. LX and LL acquired the data. LX, HS, SZ, SW, LL, XL, and YS interpreted and analyzed the data. LX and HS drafted the manuscript. SZ, SW, XL, and YS reviewed the manuscript for important intellectual content critically. All authors contributed to the article and approved the submitted version.

## Funding

This work was supported by the National Natural Science Foundation under Grant number 81922016 and 81870607, Shandong Provincial Natural Science Foundation under Grant number ZR2019JQ25, National Key R&D Program of China under Grant number 2017YFC0908900, and Innovation Fund for Outstanding Doctoral Candidates of Peking University Health Science Center (China). The funders had no role in the design and conduct of the study; collection, management, analysis, or interpretation of the data; preparation, review, or approval of the manuscript; and decision to submit the manuscript for publication.

## Conflict of Interest

The authors declare that the research was conducted in the absence of any commercial or financial relationships that could be construed as a potential conflict of interest.

## References

[1] Akchurin OM. Chronic Kidney Disease and Dietary Measures to Improve Outcomes. Pediatr Clin North Am (2019) 66(1):247–67. 10.1016/j.pcl.2018.09.007 PMC6623973 30454747

[2] National Kidney Foundation. K/DOQI Clinical Practice Guidelines for Chronic Kidney Disease: Evaluation, Classification, and Stratification. Am J Kidney Dis (2002) 39(2 Suppl 1):S1–S266. 11904577

[3] Jha V Garcia-Garcia G Iseki K Li Z Naicker S Plattner B. Chronic Kidney Disease: Global Dimension and Perspectives. Lancet (2013) 382(9888):260–72. 10.1016/S0140-6736(13)60687-X 23727169

[4] GBD Chronic Kidney Disease Collaboration. Global, Regional, and National Burden of Chronic Kidney Disease, 1990-2017: A Systematic Analysis for the Global Burden of Disease Study 2017. Lancet (2020) 395(10225):709–33. 10.1016/S0140-6736(20)30045-3 PMC7049905 32061315

[5] Srivastava A Kaze AD McMullan CJ Isakova T Waikar SS. Uric Acid and the Risks of Kidney Failure and Death in Individuals With Ckd. Am J Kidney Dis (2018) 71(3):362–70. 10.1053/j.ajkd.2017.08.017 PMC5828916 29132945

[6] Liu X Zhai T Ma R Luo C Wang H Liu L. Effects of Uric Acid-Lowering Therapy on the Progression of Chronic Kidney Disease: A Systematic Review and Meta-Analysis. Ren Fail (2018) 40(1):289–97. 10.1080/0886022X.2018.1456463 PMC6014338 29619870

[7] Kuwabara M Hisatome I Niwa K Bjornstad P Roncal-Jimenez CA Andres-Hernando A. The Optimal Range of Serum Uric Acid for Cardiometabolic Diseases: A 5-Year Japanese Cohort Study. J Clin Med (2020) 9(4):942. 10.3390/jcm9040942 PMC7231289 32235468

[8] Obermayr RP Temml C Gutjahr G Knechtelsdorfer M Oberbauer R Klauser-Braum R. Elevated Uric Acid Increases the Risk for Kidney Disease. J Am Soc Nephrol (2008) 19(12):2407–13. 10.1681/ASN.2008010080 PMC2588108 18799720

[9] Toda A Yuko I Tani M Yamakado M. Hyperuricemia is a Significant Risk Factor for the Onset of Chronic Kidney Disease. Nephron Clin Pract (2014) 126(1):33–8. 10.1159/000355639 24434843

[10] Zhu P Liu Y Han L Xu G Ran J. Serum Uric Acid Is Associated With Incident Chronic Kidney Disease in Middle-Aged Populations: A Meta-Analysis of 15 Cohort Studies. PloS One (2014) 9(6):e100801. 10.1371/journal.pone.0100801 24959886 PMC4069173

[11] Xia X Luo Q Li B Lin Z Yu X Huang F. Serum Uric Acid and Mortality in Chronic Kidney Disease: A Systematic Review and Meta-Analysis. Metabolism (2016) 65(9):1326–41. 10.1016/j.metabol.2016.05.009 27506740

[12] Saito T Sakura T Kondo K Nakamura R Oguro T Yamagami K. Serum Uric Acid and the Renin-Angiotensin System in Hypertension. J Am Geriatr Soc (1978) 26(6):241–7. 10.1111/j.1532-5415.1978.tb02396.x 659766

[13] Okada R Yasuda Y Tsushita K Wakai K Hamajima N Matsuo S. Glomerular Hyperfiltration in Prediabetes and Prehypertension. Nephrol Dial Transpl (2011) 27(5):1821–5. 10.1093/ndt/gfr651 22140135

[14] Chien KL Chen MF Hsu HC Chang WT Su TC Lee YT. Plasma Uric Acid and the Risk of Type 2 Diabetes in a Chinese Community. Clin Chem (2008) 54(2):310–6. 10.1373/clinchem.2007.095190 18089655

[15] Grayson PC Kim SY LaValley M Choi HK. Hyperuricemia and Incident Hypertension: A Systematic Review and Meta-Analysis: Risk of Incident Hypertension Associated With Hyperuricemia. Arthritis Care Res (2011) 63(1):102–10. 10.1002/acr.20344 PMC3016454 20824805

[16] Yamagata K Ishida K Sairenchi T Takahashi H Ohba S Shiigai T. Risk Factors for Chronic Kidney Disease in a Community-Based Population: A 10-Year Follow-Up Study. Kidney Int (2007) 71(2):159–66. 10.1038/sj.ki.5002017 17136030

[17] Alberti KG Eckel RH Grundy SM Zimmet PZ Cleeman JI Donato KA. Harmonizing the Metabolic Syndrome: A Joint Interim Statement of the International Diabetes Federation Task Forceon Epidemiology and Prevention; National Heart, Lung, and Blood Institute; American Heart Association; World HeartFederation; International Atherosclerosis Society; and International Association for the Study of Obesity. Obes Metab (2010) 7(1):63–5. 10.14341/2071-8713-5281 19805654

[18] Zhao Y Hu Y Smith JP Strauss J Yang G. Cohort Profile: The China Health and Retirement Longitudinal Study (CHARLS). Int J Epidemiol (2012) 43(1):61–8. 10.1093/ije/dys203 PMC3937970 23243115

[19] Wang S Shu Z Tao Q Yu C Zhan S Li L. Uric Acid and Incident Chronic Kidney Disease in a Large Health Check-Up Population in Taiwan: Uric Acid and Incident CKD. Nephrology (2011) 16(8):767–76. 10.1111/j.1440-1797.2011.01513.x 21854506

[20] Inker LA Schmid CH Tighiouart H Eckfeldt JH Feldman HI Greene T. Estimating Glomerular Filtration Rate From Serum Creatinine and Cystatin C. N Engl J Med (2012) 367(1):20–9. 10.1056/NEJMoa1114248 PMC4398023 22762315

[21] Mwasongwe SE Fülöp T DPhil RK Musani SK Sims M Correa A. Relation of Uric Acid Level to Rapid Kidney Function Decline and Development of Kidney Disease: The Jackson Heart Study. J Clin Hypertens (2018) 20(4):775–83. 10.1111/jch.13239 PMC6022371 29450959

[22] Cao X Wu L Chen Z. The Association Between Elevated Serum Uric Acid Level and an Increased Risk of Renal Function Decline in a Health Checkup Cohort in China. Int Urol Nephrol (2017) 50(3):517–25. 10.1007/s11255-017-1732-6 29094330

[23] Chen C Lu FC. The Guidelines for Prevention and Control of Overweight and Obesity in Chinese Adults. BioMed Environ Sci (2004) 17:Suppl:S1–S36. 15807475

[24] Baron RM Kenny DA. The Moderator-Mediator Variable Distinction in Social Psychological Research: Conceptual, Strategic, and Statistical Considerations. J Pers Soc Psychol (1986) 51(6):1173–82. 10.1037//0022-3514.51.6.1173 3806354

[25] Ulrich K Kristian B Anders H. Comparing Coefficients of Nested Nonlinear Probability Models. Stata J (2011) 11(3):420–38. 10.1177/1536867X1101100306

[26] Mazzali M Hughes J Kim YG Jefferson JA Kang DH Gordon KL. Elevated Uric Acid Increases Blood Pressure in the Rat by a Novel Crystal-Independent Mechanism. Hypertension (2001) 38(5):1101–6. 10.1161/hy1101.092839 11711505

[27] Kang DH Nakagawa T Feng L Watanabe S Han L Mazzali M. A Role for Uric Acid in the Progression of Renal Disease. J Am Soc Nephrol (2002) 13(12):2888–97. 10.1097/01.asn.0000034910.58454.fd 12444207

[28] Kang DH Chen W. Uric Acid and Chronic Kidney Disease: New Understanding of an Old Problem. Semin Nephrol (2011) 31(5):447–52. 10.1016/j.semnephrol.2011.08.009 22000652

[29] Ryu ES Kim MJ Shin HS Jang YH Choi HS Jo I. Uric Acid-Induced Phenotypic Transition of Renal Tubular Cells as a Novel Mechanism of Chronic Kidney Disease. Am J Physiol-Ren Physiol (2013) 304(5):F471–80. 10.1152/ajprenal.00560.2012 23283992

[30] Zeisberg M Kalluri R. The Role of Epithelial-to-Mesenchymal Transition in Renal Fibrosis. J Mol Med (2004) 82(3):175–81. 10.1007/s00109-003-0517-9 14752606

[31] Ali AA Lami FHA. Prevalence and Determinants of Microalbuminurea Among Type 2 Diabetes Mellitus Patients, Baghdad, Iraq, 2013. Saudi J Kidney Dis Transpl (2016) 27(2):348–55. 10.4103/1319-2442.178561 26997390

[32] Xia F Liu G Shi Y Zhang Y. Impact of Microalbuminuria on Incident Coronary Heart Disease, Cardiovascular and All-Cause Mortality: A Meta-Analysis of Prospective Studies. Int J Clin Exp Med (2015) 8(1):1–9. 25784968 PMC4358423

[33] Viazzi F Leoncini G Vercelli M Deferrari G Pontremoli R. Serum Uric Acid Levels Predict New-Onset Type 2 Diabetes in Hospitalized Patients With Primary Hypertension: The MAGIC Study. Diabetes Care (2011) 34(1):126–8. 10.2337/dc10-0918 PMC3005465 20921214

[34] Babio N Martínez-González MA Estruch R Wärnberg J Recondo J Ortega-Calvo M. Associations Between Serum Uric Acid Concentrations and Metabolic Syndrome and its Components in the PREDIMED Study. Nutr Metab Cardiovasc Dis (2015) 25(2):173–80. 10.1016/j.numecd.2014.10.006 25511785

[35] Khosla UM Zharikov S Finch JL Nakagawa T Roncal C Mu W. Hyperuricemia Induces Endothelial Dysfunction. Kidney Int (2005) 67(5):1739–42. 10.1111/j.1523-1755.2005.00273.x 15840020

[36] Lv Q Meng XF He FF Chen S Su H Xiong J. High Serum Uric Acid and Increased Risk of Type 2 Diabetes: A Systemic Review and Meta-Analysis of Prospective Cohort Studies. PloS One (2013) 8(2):e56864. 10.1371/journal.pone.0056864 23437258 PMC3577701

[37] Wang L Zhang T Liu Y Tang F Xue F. Association of Serum Uric Acid With Metabolic Syndrome and its Components: A Mendelian Randomization Analysis. BioMed Res Int (2020) 2020:6238693. 10.1155/2020/6238693 32258131 PMC7063870

[38] Li L Song Q Yang X. Lack of Associations Between Elevated Serum Uric Acid and Components of Metabolic Syndrome Such as Hypertension, Dyslipidemia, and T2DM in Overweight and Obese Chinese Adults. J Diabetes Res (2019) 2019:3175418. 10.1155/2019/3175418 31871945 PMC6913180

[39] Helal I Fick-Brosnahan GM Reed-Gitomer B Schrier RW. Glomerular Hyperfiltration: Definitions, Mechanisms and Clinical Implications. Nat Rev Nephrol (2012) 8(5):293–300. 10.1038/nrneph.2012.19 22349487

[40] Okada R Wakai K Naito M Morita E Kawai S Yin G. Renal Hyperfiltration in Prediabetes Confirmed by Fasting Plasma Glucose and Hemoglobin A1c. Ren Fail (2012) 34(9):1084–90. 10.3109/0886022X.2012.717516 22978359

[41] Brenner BM Lawler EV Mackenzie HS. The Hyperfiltration Theory: A Paradigm Shift in Nephrology. Kidney Int (1996) 49(6):1774–7. 10.1038/ki.1996.265 8743495

[42] Krikken JA Gansevoort RT Dullaart RPF. On Behalf of the PREVEND Study Group. Lower HDL-C and Apolipoprotein A-I are Related to Higher Glomerular Filtration Rate in Subjects Without Kidney Disease. J Lipid Res (2010) 51(7):1982–90. 10.1194/jlr.M005348 PMC2882715 20211930

[43] Kuma A Uchino B Ochiai Y Kawashima M Enta K Tamura M. Impact of Low-Density Lipoprotein Cholesterol on Decline in Estimated Glomerular Filtration Rate in Apparently Healthy Young to Middle-Aged Working Men. Clin Exp Nephrol (2018) 22(1):15–27. 10.1007/s10157-017-1407-8 28386655

[44] Liang X Ye M Tao M Zheng D Cai R Zhu Y. The Association Between Dyslipidemia and the Incidence of Chronic Kidney Disease in the General Zhejiang Population: A Retrospective Study. BMJ Nephrol (2020) 21(1):252. 10.1186/s12882-020-01907-5 PMC7330963 32616008

[45] Vaziri ND. Dyslipidemia of Chronic Renal Failure: The Nature, Mechanisms, and Potential Consequences. Am J Physiol-Ren Physiol (2006) 290(2):F262–72. 10.1152/ajprenal.00099.2005 16403839

[46] Zewinger S Speer T Kleber ME Scharnagl H Woitas R Lepper PM. Hdl Cholesterol Is Not Associated With Lower Mortality in Patients With Kidney Dysfunction. J Am Soc Nephrol (2014) 25(5):1073–82. 10.1681/ASN.2013050482 PMC4005298 24610925

[47] Bash LD Coresh J Köttgen A Parekh RS Fulop T Wang Y. Defining Incident Chronic Kidney Disease in the Research Setting: The Aric Study. Am J Epidemiol (2009) 170(4):414–24. 10.1093/aje/kwp151 PMC2727177 19535543

[48] Cain L Shankar A Ducatman AM Steenland K. The Relationship Between Serum Uric Acid and Chronic Kidney Disease Among Appalachian Adults. Nephrol Dial Transpl (2010) 25(11):3593–9. 10.1093/ndt/gfq262 PMC2980994 20501458

[49] Yuan X Liu T Wu L Zou ZY Li C. Validity of Self-Reported Diabetes Among Middle-Aged and Older Chinese Adults: The China Health and Retirement Longitudinal Study. BMJ Open (2015) 5(4):e006633. 10.1136/bmjopen-2014-006633 PMC4401856 25872937

[50] Martin LM Leff M Calonge N Garrett C Nelson DE. Validation of Self-Reported Chronic Conditions and Health Services in a Managed Care Population. Am J Prev Med (2000) 18(3):215–8. 10.1016/s0749-3797(99)00158-0 10722987

